# Food Price Spikes Are Associated with Increased Malnutrition among Children in Andhra Pradesh, India^1^,^2^,^3^

**DOI:** 10.3945/jn.115.211250

**Published:** 2015-07-01

**Authors:** Sukumar Vellakkal, Jasmine Fledderjohann, Sanjay Basu, Sutapa Agrawal, Shah Ebrahim, Oona Campbell, Pat Doyle, David Stuckler

**Affiliations:** 4 Department of Sociology, University of Oxford, Oxford, United Kingdom;; 5 Department of Medicine, Stanford University, Stanford, CA;; 6 Public Health Foundation of India, New Delhi, India; and; 7 Department of Non-Communicable Disease Epidemiology, London School of Hygiene and Tropical Medicine, London, United Kingdom

**Keywords:** food price spikes, food consumption, weight-for-height *z* scores, child nutrition, India

## Abstract

**Background:** Global food prices have risen sharply since 2007. The impact of food price spikes on the risk of malnutrition in children is not well understood.

**Objective:** We investigated the associations between food price spikes and childhood malnutrition in Andhra Pradesh, one of India’s largest states, with >85 million people. Because wasting (thinness) indicates in most cases a recent and severe process of weight loss that is often associated with acute food shortage, we tested the hypothesis that the escalating prices of rice, legumes, eggs, and other staples of Indian diets significantly increased the risk of wasting (weight-for-height *z* scores) in children.

**Methods:** We studied periods before (2006) and directly after (2009) India’s food price spikes with the use of the Young Lives longitudinal cohort of 1918 children in Andhra Pradesh linked to food price data from the National Sample Survey Office. Two-stage least squares instrumental variable models assessed the relation of food price changes to food consumption and wasting prevalence (weight-for-height *z* scores).

**Results:** Before the 2007 food price spike, wasting prevalence fell from 19.4% in 2002 to 18.8% in 2006. Coinciding with India’s escalating food prices, wasting increased significantly to 28.0% in 2009. These increases were concentrated among low- (χ^2^: 21.6, *P* < 0.001) and middle- (χ^2^: 25.9, *P* < 0.001) income groups, but not among high-income groups (χ^2^: 3.08, *P* = 0.079). Each 10.0 rupee ($0.170) increase in the price of rice/kg was associated with a drop in child-level rice consumption of 73.0 g/d (β: −7.30; 95% CI: −10.5, −3.90). Correspondingly, lower rice consumption was significantly associated with lower weight-for-height *z* scores (i.e., wasting) by 0.005 (95% CI: 0.001, 0.008), as seen with most other food categories.

**Conclusion:** Rising food prices were associated with an increased risk of malnutrition among children in India. Policies to help ensure the affordability of food in the context of economic growth are likely critical for promoting children’s nutrition.

## Introduction

Globally, about one-third of all malnourished children live in India. Although India has experienced remarkable economic growth since 2000 (1), the nation’s progress in reducing malnutrition has stagnated (2, 3). According to the latest round of the National Family Health Survey for the years 2005–2006, ∼16.0% of Indian children suffered from wasting and 50.0% were underweight, with little improvement in these statistics over the past decade (4).

One hypothesis for the failure of India’s nutrition to keep pace with economic development is that the poor remain vulnerable to spikes in food prices. Although the reasons for the recent spike are debated, scarcity of food is one fundamental problem. A combination of short- and long-term factors play a role, including adverse weather conditions, higher fertilizer and fuel prices, growing population and growing meat demand (5), diversion of agricultural land for feedstock crops driven by biofuel production, and stagnation in investments in agricultural production technology (6–8). Shortfalls in food supplies and increases in domestic prices in producing countries may have triggered a chain reaction of trade-curtailing policies and panic buying, resulting in further pressure on the international market (6). For example, worldwide, food prices rose sharply in the aftermath of the 2007 financial crisis (9). The FAO’s Global Food Price Index jumped from 135 in January 2007 to 226 in June 2008 (10); prices of bananas rose by 31.0%, wheat by 77.0%, and rice by 166% (11). Ecological projections have suggested that these price rises would tip an additional 75.0 million people into extreme hunger (7, 12).

Previous studies have outlined the pathways of the effects of rising food prices on micronutrient intake and malnutrition outcomes in children (13, 14), and have argued using simulation regression models that these spikes food prices may have a widespread adverse impact on maternal and child health, especially in low- and middle-income countries (13, 15–20). Observational work in neighboring China has also documented the role of rising food prices in shaping macronutrient consumption, pointing to the importance of a carefully planned combination of taxes and subsidies to change dietary patterns in the face of the nutrition transition (21, 22). Similar observational findings on the role of food prices in shaping consumption patterns, food security, and malnutrition have been documented in other settings, e.g., in the United States—particularly among low-income households (23, 24)—Guatemala (14), and Burkina Faso in the period after the 2008 food price spike (19). Taken together, this literature suggests that, even when families are able to employ resilience strategies by increasing food expenditures or engaging in food substitutions, sharp rises in food prices may result in caloric deficits, particularly for low-income groups.

**FIGURE 1:**
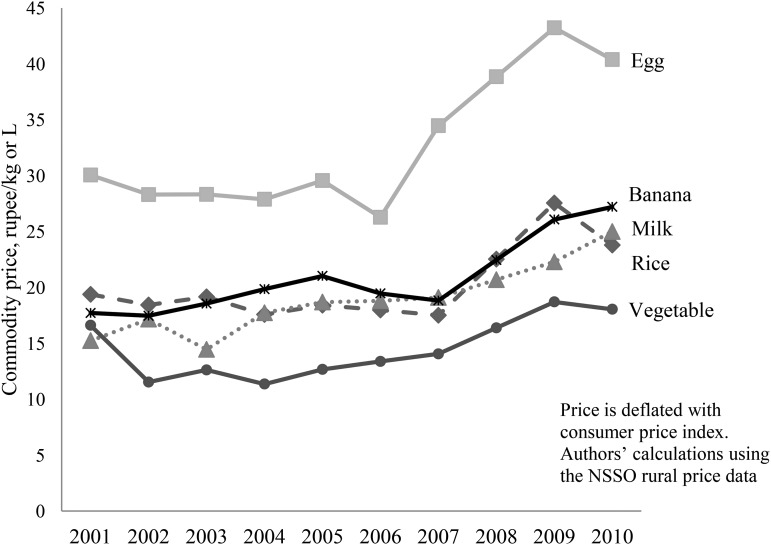
Food price trends in Andhra Pradesh, India, 2001–2010. NSSO, National Sample Survey Office.

Testing the impact of food price spikes on the nutrition of Indian children is challenging, in part because of the lack of available longitudinal data that link children’s food consumption to market environments. Our initial review of the monthly rural price dataset from the Indian National Sample Survey Office (NSSO)^8^ reveals that domestic food prices did increase in association with global rises in food commodity prices, albeit less markedly than the global trend, between 2006 and 2009 (25). In India, real domestic food prices rose slightly between 2002 and 2006, but increased sharply after 2007 (**Figure 1**). Between 2006 and 2009, real prices increased for milk (21.6%), meat (23.8%), rice (36.8%), vegetables (56.5%), legumes (70.0%), and eggs (81.2%).

In the current study, we undertook the first direct test of the hypothesis that the escalating prices of rice, legumes, eggs, and other staples of Indian diets significantly increased the risk of wasting in children by linking food price data with longitudinal data on children’s food consumption and nutritional outcomes from the Young Lives India data set, allowing us to examine within-child changes. The Young Lives data set is unique because it follows children over time from birth through age 8 y. Two-stage least squares (2SLS) models were used to test the joint hypotheses that rising food prices reduced children’s food consumption and that the associated reduction in consumption increased the risk of wasting.

## Methods

### 

#### Study settings.

We used data covering periods before and after food price spikes in the state of Andhra Pradesh, one of India’s largest states, with a population of >85 million people (26). Formal ethical approval for the Young Lives study was obtained from independent ethics committees at the London School of Hygiene, London South Bank University, and University of Reading, United Kingdom (27). The publicly available data did not contain any personal identifiers; new ethical approval was therefore not necessary for fully anonymized secondary data analysis.

#### Measuring children’s food consumption and wasting.

Data on children’s food consumption and nutrition were taken from the Young Lives study in combination with the data from the NSSO rural price survey (25), NSSO consumer expenditure surveys (28, 29) and National Nutrition Monitoring Bureau (NNMB) surveys (30, 31) in Andhra Pradesh. Young Lives is a longitudinal cohort study set up in 7 districts of Andhra Pradesh. The initial sample included 2000 children aged 6–18 mo in 2002 (wave 1), followed up at ∼5 y of age in 2006 (wave 2), and again at 8 y of age in 2009 (wave 3). Sampling details of the Young Lives study have been described elsewhere (27, 32) Briefly, data that are representative of Andhra Pradesh were collected for all socioeconomic groups, drawing from the 3 major geographic locations in Andhra Pradesh (Seemandhra, Telengana, and Rayalaseema), each including a socioeconomically developed site and nondeveloped site. The survey collected information on a wide range of household-, policy-, and child poverty–related variables from caregivers. Attrition rates and missing data were low: 4.00% of the sample was lost to follow-up, an additional 8 cases lacked nutrition measures, and 4 cases were deleted as outliers, having standardized residuals greater than |2| for the food consumption data, yielding a final analytic sample of *n* = 1918 children, which yields sufficient power to detect a 6.00% change in dietary intake among food items with 80.0% power. Wave 2 data were used to define income in our study. Our analysis was primarily based on data from waves 2 and 3, because the consumption data were not available in wave 1, and data from wave 1 were used only in the descriptive analysis highlighting changes in child malnutrition over time.

Children’s food intake was measured in waves 2 and 3 by recording the household food expenditure for the past 15 d from the report from the caretaker (usually the mother) obtained through personal interview. Food items were aggregated into 8 major consumption categories: rice, legumes (including, e.g., dried peas, edible beans, lentils, and chickpeas), meat (including chicken, goat, and lamb), fish, eggs, milk, fruit, and vegetables. Daily food consumption was quantified by merging the NSSO consumer expenditure survey of household food purchasing with the Young Lives data. Each household in the NSSO data reported the amount (in kilograms) and the price (in rupees per kilogram) purchased in the last 15 d for 132 food items. With the use of the NSSO data, weighted means were constructed for each of the 8 broad food categories used in the Young Lives data. Within each food category, weights were based on the proportion of each food item consumed. For example, within the category of legumes, a household in the Young Lives data may have reported consuming moong dal, chana dal, and gram dal; the price paid for each separate type of dal was also reported. Total household expenditure on legumes was then averaged across weighted prices and separately among low, middle, and high income groups for each food category. Because the estimated food consumption data were at the household level, we further multiplied this consumption with the use of the weighted mean of the child-level consumption that was computed from the NNMB data from Andhra Pradesh (30, 31). The NNMB collected data on household level and individual level for different age groups in the years 2001 and 2011–2012. Because food price spikes might change within-household allocation of food, we used the year 2001 consumption level of age 4–6 y in the prefood price spike period of wave 2 data, and the year 2011–2012 consumption level of age 7–9 y in the postfood spike period of wave 3 data. Following WHO standards (33), Young Lives calculated children’s nutrition outcomes with the use of standardized *z* score measures that compare children’s weight, height, and age with the WHO reference population distribution, including wasting (weight-for-height), which is susceptible to short-term fluctuations, and, as a control condition, stunting (height-for-age), because height should not change rapidly in response to a food price spike (4, 33). These *z* scores have the same statistical relation to the distribution of the reference around the mean at all ages, which makes results comparable across ages, groups, and populations (33). Wasting (thinness) is a strong predictor of infant mortality, indicating in most cases a recent and severe process of weight loss that is often associated with acute food shortage (4, 33). We evaluated *z* scores as continuous variables, and conducted analyses of a dichotomous indicator of wasting (i.e., weight-for-age <−2 SDs from the mean for the reference population) as a consistency check.

#### Measuring food prices.

Food price data were taken from the NSSO monthly price records collected by the Government of India, covering 132 food commodities from 603 village market hubs spread over 24 major Indian states. District-level food price data from the 7 districts of Andhra Pradesh were included in Young Lives. To correct for nonfood price inflation (and thereby measure purchasing power), we adjusted the data with the use of the government-approved nonfood consumer price index (34).

#### Statistical analysis.

The 2SLS models were used first to assess the association between changes in food prices and food consumption, and then to assess the relation between these consumption changes and children’s nutritional risk of malnutrition. The “ivreg” command in STATA, which simultaneously corrects the coefficients and SEs for the 2-stage design, was applied to fit the 2SLS difference models. We used food price as our instrument because nutrition is endogenous to food consumption, but food prices are exogenously related to child nutrition. The instrumental variable, i.e., food price, is not directly correlated with the wasting, but rather operates through food consumption. This instrumental variable approach substantially reduces potential confounding between nutrition and consumption because any unobserved endogenous factors would need to be associated with both food prices and food consumption, which is unlikely, because there were no major natural events period (such as droughts or floods) during the study in Andhra Pradesh. We adjusted for potential confounding sociodemographic factors, including children’s age, sex, rural/urban residence, income, and mother’s education, as follows:


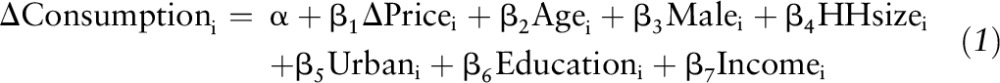



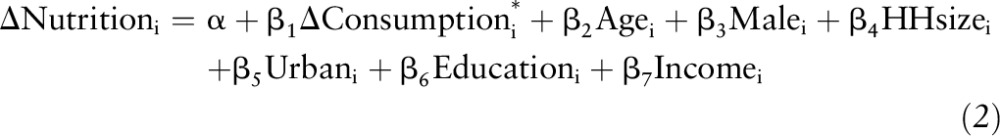


where *i* is child; Δ is the difference in value in 2009 compared with the year 2006; consumption is a vector of per capita daily food consumption, and the asterisk denotes the prediction from the first-stage equation; nutrition is children’s weight-for-height *z* score (wasting); price is a vector of food prices for each of the 8 food items; HHsize is the household size; urban is a dummy for the household’s urban or rural location; education is the mother’s educational years; and income is a categorical variable of 3 evenly divided income groups (low-, middle-, and high-income). The α for all models was α = 0.05. Values in the text are means ± 95% CIs, whereas values in the tables are means ± SEMs. All models were performed with the use of STATA version 13.1 (35).

## Results

### 

#### Rising food prices and consumption.

Descriptive statistics for the analytic sample are provided in **Table 1**. Fifty-three percent of children were male. The mean age was 12.3 mo in wave 1, 64.8 mo in wave 2 and 96.0 mo in wave 3.

**TABLE 1:** Sample characteristics of 1918 Indian children in the Young Lives study, Andhra Pradesh, India^1^

	Wave 1, 2002	Wave 2, 2006	Wave 3, 2009
Children, *n*	1918	1918	1918
Age, mo	12.3 ± 3.50	64.8 ± 3.80	96.0 ± 3.90
Male	53.0	53.0	53.0
Urban	24.1	25.3	26.1
Mother’s education			
Illiterate	51.1	NA	NA
Lower primary school	10.2	NA	NA
Upper primary school	16.0	NA	NA
Secondary/higher secondary	19.3	NA	NA
College/university	3.20	NA	NA
Ethnicity/caste		NA	NA
Mixed caste	0.100	NA	NA
Schedule caste	17.9	NA	NA
Schedule tribe	13.0	NA	NA
Backward caste	47.9	NA	NA
Other caste	20.9	NA	NA
Childhood nutrition			
Weight-for-height *z* score	−1.00 ± 1.20	−1.20 ± 1.00	−1.40 ± 1.20
Height-for-age *z* score	−1.30 ± 1.60	−1.60 ± 1.10	−1.40 ± 1.20
Wasting prevalence	19.4	18.8	28.0
Stunting prevalence	30.1	35.8	29.2
Children’s food intake			
Rice, g/d	NA	226 ± 293	193 ± 143
Legumes, g/d	NA	16.0 ± 19.0	14.0 ± 12.0
Meat, g/d	NA	2.00 ± 4.00	6.00 ± 6.00
Eggs, g/d	NA	2.00 ± 2.00	5.00 ± 6.00
Fish, g/d	NA	1.00 ± 2.00	2.00 ± 4.00
Milk, mL/d	NA	57.0 ± 79.0	62.0 ± 74.0
Vegetables, g/d	NA	10.0 ± 6.00	7.00 ± 4.00
Fruits, g/d	NA	10.0 ± 15.0	8.00 ± 11.0
Food prices			
Rice, rupees/kg	16.5 ± 1.20	17.4 ± 1.90	23.8 ± 2.7
Legumes, rupees/kg	41.1 ± 1.50	46.1 ± 3.30	78.3 ± 2.8
Meat, rupees/kg	136 ± 14.9	140 ± 8.30	173 ± 14.3
Fish, rupees/kg	68.9 ± 41.3	75.0 ± 45.8	88.8 ± 34.7
Eggs, rupees/kg	28.0 ± 0.700	26.0 ± 1.90	47.1 ± 9.50
Milk, rupees/L	19.8 ± 2.50	17.1 ± 1.90	20.8 ± 4.60
Vegetables, rupees/kg	11.2 ± 0.700	12.4 ± 1.10	19.4 ± 2.60
Fruits, rupees/kg	19.3 ± 2.80	21.5 ± 3.50	28.9 ± 2.20

1 Values are means ± SDs or percentages. Food prices were in constant prices, adjusted for inflation based on the Government of India’s estimates for nonfood consumer price index. NA, not available.

In the sample, children’s food consumption dropped significantly between 2006 and 2009 as food prices increased. The reported daily food consumption for rice fell from 226 g/d to 193 g/d, legumes from 16.0 g/d to 14.0 g/d, vegetables from 10.0 g/d to 7.00 g/d, and fruits from 10.0 g/d to 7.00 g/d (Table 1). In contrast, however, milk and meat consumption increased, from 57.0 mL/d to 62.0 mL/d and 2.00 g/d to 6.00 g/d, respectively. These declines were relatively heterogeneous across income groups, but create greater absolute nutritional risks of malnutrition among low-income children. In 2009, rice consumption among children in the low-income tertile dropped 28.0%, from 203 g/d to 146 g/d, whereas in the high-income tertile, the corresponding decrease was 5.00%, from 242 g/d to 230 g/d (**Supplemental Table 1**). Low-income groups also experienced proportionally greater declines in the consumption of the major components of their diet, such as rice, legumes, vegetables, and fruits, but an increase in the consumption of milk and meat (**Supplemental Figure 1**).

The estimates of marginal effect of price from the first-stage regression models (**Table 2**) showed that each 10.0 rupee increase ($0.170) in the price (per kilogram) was associated with a decrease in children’s per capita daily food consumption of rice by 73.0 g/d (β: −7.30; 95% CI: −10.6, −3.90), vegetables by 5.00 g/d (β: −0.500; 95% CI: −0.600, −0.400), fruits by 2.00 g/d (β: −0.200; 95% CI:−0.300, −0.020), eggs by 1.00 g/d (β: −0.100; 95% CI: −0.110, −0.060), and meat by <1.00 g/d (β: −0.040; 95% CI: −0.060, −0.010). Furthermore, a 1.00 rupee increase in the price (per liter) of milk was associated with a 2.00 mL/d decrease (β: −2.02; 95% CI: −2.63, −1.40) in the per capita daily consumption of milk. However, although the univariate analysis showed a decrease in the consumption of legumes, there was evidence of an increase in the consumption of legumes by 2.00 g/d (β: 0.200; 95% CI: 0.070, 0.300) and fish by <1.00 g/d (β: 0.040; 95% CI: 0.010, 0.060) according to the 2SLS model.

**TABLE 2:** Association between price rises and per capita daily consumption of food and weight-for-height *z* score in the year 2009 compared with 2006 among children in Andhra Pradesh, India^1^

	Rice	Legumes	Vegetables	Fruits	Milk	Eggs	Meat	Fish
First-stage estimates^2^								
Change in price^3^	−7.25 ± 3.42***	0.180 ± 0.060***	−0.530 ± 0.050***	−0.160 ± 0.070*	−2.02 ± 0.310***	−0.090 ± 0.010***	−0.040 ± 0.010**	0.020 ± 0.007**
High-income	ref	ref	ref	ref	ref	ref	ref	ref
Middle-income	−26.0 ± 9.36**	−1.51 ± 0.780*	−0.450 ± 0.230*	−3.32 ± 0.690***	−36.8 ± 4.12***	−2.61 ± 0.350***	−1.13 ± 0.380**	−0.770 ± 0.290**
Low-income	−57.9 ± 10.4***	−3.66 ± 0.820***	−0.730 ± 0.250**	−5.03 ± 0.730***	−48.0 ± 4.38***	−3.67 ± 0.370***	−1.86 ± 0.400***	−1.97 ± 0.300***
Age	−0.190 ± 0.810	−0.120 ± 0.070	0.040 ± 0.020	−0.030 ± 0.060	0.130 ± 0.370	−0.010 ± 0.310	0.070 ± 0.030*	0.010 ± 0.030
Urban	−13.6 ± 9.24	−2.21 ± 0.780***	0.470 ± 0.240*	1.13 ± 0.690	31.1 ± 4.15***	−0.060 ± 0.350	0.480 ± 0.380	−0.910 ± 0.280***
Male	9.64 ± 6.38	0.140 ± 0.550	0.110 ± 0.160	−0.840 ± 0.490	4.07 ± 2.90	−0.200 ± 0.250	0.200 ± 0.270	0.100 ± 0.200
Household size	−6.28 ± 1.44***	−0.720 ± 0.180***	−0.420 ± 0.040***	−0.590 ± 0.110***	−3.85 ± 0.660***	−0.410 ± 0.060***	−0.260 ± 0.060***	−0.320 ± 0.050***
Second-stage estimates^4^								
Change in consumption^5^	0.005 ± 0.002*	0.080 ± 0.040*	0.070 ± 0.030*	−0.020 ± 0.050	0.004 ± 0.003	0.080 ± 0.030**	−0.300 ± 0.100*	0.100 ± 0.100
* R*^2^	0.050	0.030	0.140	0.090	0.260	0.110	0.040	0.050

1 Values are marginal effects ± SEs. Estimates of the 2-stage least squares difference model through food price as the instrumental variable. Constant estimated but not reported. *P < 0.05, **P < 0.01, ***P < 0.001. ref, reference.

2 Marginal effect of change in price of food items on the change in children’s daily food consumption (in grams or milliliters).

3 Per kilogram or liter of food item.

4 Marginal effect of change in daily food consumption (in grams or milliliters) on the change in weight-for-height *z* score.

5 In grams or milliliters per day.

#### Risks of wasting.

In parallel with these trends, before the increase in prices between 2002 and 2006, the proportion of children who suffered from wasting fell slightly, from 19.4% (95% CI: 17.6%, 21.1%) in 2002 to 18.8% (95% CI: 17.1%, 20.6%) in 2006. In the subsequent period, when food prices rose markedly, wasting rose to 28.0% (95% CI: 26.0%, 30.0%) by 2009. In contrast, consistent with the idea of a secular trend in reduced stunting, we observed a steady decline in stunting during the same period (35.8%; 95% CI: 33.6%, 38.0% in 2006 and 29.2%; 95% CI: 27.1%, 31.2% in 2009), which was unaffected by short-term food consumption and driven by long-term height determinants (i.e., requiring ≥5 y of deprivation to change); this was unassociated with the food price spikes, serving as an effective negative control and falsification test (36).

**FIGURE 2:**
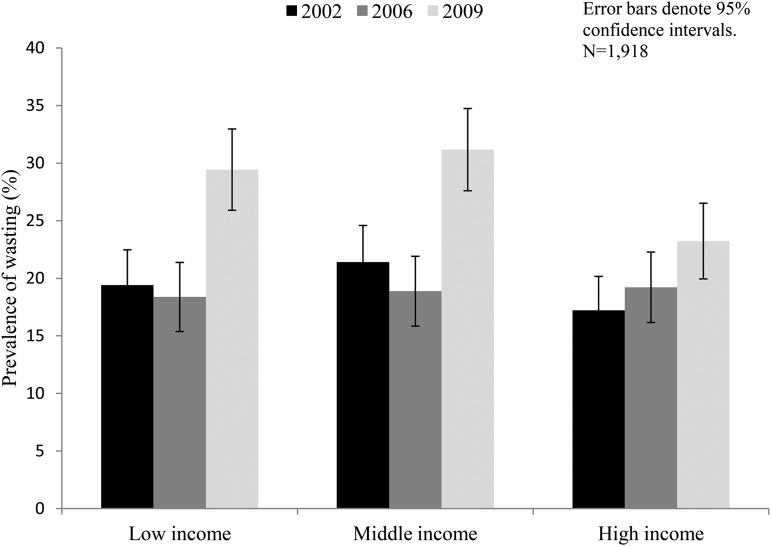
Trends in prevalence of wasting in children in Andhra Pradesh, India, by income group.

The observed increase in wasting was disproportionately greater among the low- and middle-income households than in high-income ones. On average, prevalence of wasting rose in low-income groups from 18.4% (95% CI: 15.4%, 21.4%) to 29.4% (95% CI: 25.9%, 33.0%), in middle-income households from 18.9% (95% CI: 15.8%, 21.9%) to 31.2% (95% CI: 27.6%, 34.7%), and in high-income households from 19.2% (95% CI: 16.2%, 22.3%) to 23.2% (95% CI: 19.9%, 26.5%) (**Figure 2**). These prevalence increases were statistically significant among low- and middle-income groups (χ^2^: 21.6, *P* < 0.001 and χ^2^: 25.9, *P* < 0.001, respectively) but not among the high-income groups (χ^2^: 3.08, *P* = 0.079).

In the second stage of the regression model, which quantified the association of food consumption with children’s risk of wasting (Table 2), we found greater per capita daily food consumption in children’s weight-for-height *z* score (wasting) in the year 2009 compared with 2006. Each additional per capita consumption of 1.00 g/d of rice was associated with a 0.005 unit (95% CI: 0.001, 0.009) increase in children’s weight-for-height *z* score in the year 2009 compared with 2006. Similar trends were observed for the associations with consuming legumes (β: 0.080; 95% CI: 0.010, 0.200), vegetables (β, 0.070; 95% CI: 0.010, 0.100), and eggs (β: 0.080; 95% CI: 0.020, 0.100).

#### Robustness checks.

Because children’s nutritional needs vary with age, we validated the energy intake of children at each wave by using the guidelines of the Indian Council for Medical Research (ICMR) (37), which have been widely used in setting nutrition policy in India. We calculated per-day caloric intake with the use of the ICMR’s table of caloric contents for each food item and compared this intake with the ICMR recommended minimum calorie requirements of 1944 kcal for a child aged 4–6 y and 2349 kcal for a child aged 7–9 y. We found that there was an increase in the number of children with a deficiency of the recommended minimum caloric intake from 23.0% in 2006 to 37.0% in 2009. The increase in the number of children with a deficiency in caloric intake was observed to be higher in the low-income (from 31.0% to 48.0%) and middle-income (from 25.5% to 39.0%) tertiles than in the higher-income tertile (from 16.0% to 23.0%).

Additionally, to check if the estimated child-level food intake is subject to measurement error, we reran our models with the use of per capita household-level food intake (**Supplemental Table 2**). The magnitude of the associations increased, but, on the whole, the associations between prices, consumption, and malnutrition at the household level were very similar to those at the child level. Moreover, our estimated data for per capita consumption by children, as well as household-level consumption, in the pre- and postfood price-spike periods were comparable with the consumption pattern reported in the NNMB data of 2001 and 2011–2012, as well as with household-level data from NSSO consumer expenditure surveys of 2005 and 2010 for Andhra Pradesh.

To test potential interactions with income group, we also replicated the analysis stratifying the data into 3 income groups (**Supplemental Table 3**). As expected, we found a higher marginal effect from price among the low-income and middle-income tertiles than among the high-income tertile for most food items. We also included a control variable for the length of time between interviews across waves 2 and 3 to capture the potential effects of variation in the time interval across waves. None of our main findings was affected. In addition, we performed several estimates of cross-effects of price to examine the possibility of substitution between different food items, but did not detect statistically significant associations.

## Discussion

We investigated the associations between food price spikes and children’s nutrition in India by applying a 2SLS modeling approach to a unique experiment created by recent price fluctuations. Our statistical models suggest that rising food prices are associated with negative childhood nutrition across the entire population, but particularly among deprived groups, consistent with the theory that the latter groups have the smallest food reserves and therefore are less resilient to price variations. Rising food prices were associated with significant declines in the consumption of rice, a major source of caloric intake, and eggs and meat, which are important high-protein sources in Indian diets. In turn, these declines were associated with a greater risk of children’s wasting, a short-term respondent to prices, but not stunting, which is a long-term deprivation measure. However, it is worth noting that long-term decreases in stunting are often the result of a data artifact, whereby substantial recovery from stunting occurs between mid-childhood and adulthood—a phenomenon typically unrelated to any nutritional interventions or the income status of the parents (38–40). However, even if improvements in child malnutrition are associated with critical growth windows in the absence of nutritional interventions, such interventions may improve further child health outcomes, and early childhood interventions may have important but yet-unknown implications for malnutrition and growth later in childhood and adolescence (41).

As with any statistical modeling analysis, our study has several limitations. First, the study is not representative of the entirety of India because of the lack of nationally representative, longitudinal data on children’s nutrition during the period of interest. However, Andhra Pradesh is a large state of 85.0 million people (26), and it reflects diversity in income ranges typical of Indian states (42). Andhra Pradesh is approximately in the middle of all Indian states in terms of socioeconomic and health indicators (43), and our data included representative data on all socioeconomic groups in the state. It is likely that the patterns observed here would apply to other Indian states, because household food expenditure would be susceptible to food price changes. This is corroborated by the observation, with the use of nationally representative data, that the proportion of food expenditure to total household expenditure in the country increased from 48.8% in 2005 to 50.7% in 2010, in contrast to historically declining trends (28, 29). However, this analysis is likely to understate the magnitude of wasting in association with food price rises because difficult-to-reach populations, such as persons living in slums, may not be fully captured by the Young Lives survey. Although there was a low attrition rate (4.00%), the few cases that dropped out of the survey were likely to be at higher risk of deteriorating nutrition over the study period. Second, we were constrained in providing an analysis on the real variability within the sample of each income category, mainly because the data set we used only had a categorical variable of classifying children to income tertile, instead of the actual income as continuous variable.

Third, as with any econometric assessment, there is the potential for unobserved or unmeasured confounders. We attempted to reduce this risk with the use of a 2SLS approach, such that confounders would have to be related both to spikes on food prices and to nutritional outcomes, which is unlikely, given the known causes of the food spikes in terms of futures market trading (44). Furthermore, the 2SLS design is only consistent if the instrument (in this case food prices) is independent of the error term in the main equation predicting anthropometric status. Any association between price and nutrition status that is not through food consumption may confound the results (these include, e.g., weather, climate change, environmental degradation, recession, and political turmoil, which can simultaneously affect prices and nutrition status). However, to our knowledge, there were no major climatic events, political turmoil, or other external factors of this nature that would systematically bias the link between food prices and food consumption. Additionally, we estimated the ordinary least squares model of consumption on wasting and found that the use of the 2SLS model enabled us to predict the effect of price on wasting (**Supplemental Table 4**). Although an experimental design would be preferable for testing potential causal associations, there are serious practical and ethical issues with such a design. However, future research should consider the possibility of using randomized controlled trials as a means of further testing the associations documented here. Fourth, our study lacked sufficient statistical power to fully investigate substitution effects across food groups. For instance, it is likely that the observed increased consumption of milk and vegetables helped offset a further worsening in wasting during food price rises, as families switched to more affordable food items rather than forgoing these calories altogether. The issue of food substitution itself is another important research theme to be rigorously examined in subsequent studies that use larger data sets. Future research is needed to better understand resilience strategies that households and communities may employ to smooth food consumption in the face of economic shocks.

Fifth, although standardized *z* scores against WHO reference populations were used to adjust directly for aging and models indirectly corrected for aging, it is nonetheless possible that adjusting for aging may have biased results. Such bias, however, is likely to be nondifferential with respect to associations of food price fluctuations, thereby yielding conservative estimates. Our analysis also did not reveal significant *z* score changes when children aged from ∼2 y to 6 y from 2002 to 2006. Related to this point, the *z* scores of the children between waves 2 and 3 might not be directly comparable, because the standard *z* scores of the children in wave 2 were based on the WHO reference population of 0–5 y of age, whereas the *z* scores of the children in wave 3 were based on the reference population of 5–18 y of age. However, the hypothesized relation between food prices and nutrition is unlikely to be affected by this concern, because any potential bias arising from this concern would be nondifferential. According to the WHO, “…*z* scores have the same statistical relation to the distribution of the reference around the mean at all ages, which makes results comparable across age groups and indicators” (45). Thus, to our knowledge, WHO *z* scores are the best standardized metrics for tracking child malnutrition (46–48). Finally, because our measure of consumption was based on caretaker reports of food expenditure across a 2 wk period, parent-provided recall bias is possible, because precise and accurate measurement of exact food intake is difficult. For caretaker reports (for young children), it is also possible that older children obtained additional food from other sources, such as free school lunches provided as part of India’s midday meal scheme. Similarly, because consumption is estimated based on food expenditures rather than directly observed, there may be some imprecision in our consumption measurement. However, we have no reason to believe that these potential sources of bias would be systematic in relation to food price fluctuations so as to bias our findings qualitatively.

Our study has important implications for future research on how to design policies to improve nutrition. First, identifying which policies may mitigate the transfer of global price spikes into local price spikes on food may be critical for maintaining nutrition. Second, we observed overall declines in food intake, particularly among the poor, but no study based on observed dietary recalls can determine which food groups are being substituted for which others. Hence, further study of substitution effects during spikes may help identify the sources of resilience among the poor and what foods are preferentially consumed during spikes, which has implications for micronutrient deficiencies.

World Bank economists predict that, although food prices will fluctuate, prices will remain elevated until at least 2015 (49). This suggests that it remains important to address food prices even as attention to recent price spikes has dissipated. It is critical to implement policy measures to control the root causes of food price spikes. This may involve improving irrigation facilities, increasing agriculture production through better seeds and other technological measures, strengthening the supply chains to minimize the rotting of food grains, and diversifying the production base (50). Moreover, household resilience strategies may be improved by identification of and public education about food item substitutions that offer the most nutritional value for the lowest possible cost—a process that may be facilitated by nutrient profiling focused on staple foods in the Indian diet (51). India’s passage of the 2013 National Food Security Act and similar provisions in other countries offer researchers opportunities to prospectively identify how nutrition programs may alter the risk of poor nutrition (52, 53). Our study implies that not only average annual food consumption, but temporal nutritional changes associated with acute food price spikes, should be evaluated to ensure nutritional adequacy as such policies and programs are introduced.

## Footnotes

8 Abbreviations used: ICMR, Indian Council for Medical Research; NNMB, National Nutrition Monitoring Bureau; NSSO, National Sample Survey Office; 2SLS, two-stage least squares.
